# Correspondence

**Published:** 1866-05

**Authors:** James P. White

**Affiliations:** Madrid, Spain


### Correspondence.

MADRID, SPAIN, February 12, 1866.

To the Editor of the Buffalo Medical and Surgical Journal:

*My Dear Doctor:*—When last I wrote you we were about to depart for Spain. Our route lay through Orleans, Tours, Bordeaux, and Boyone, whence we took the western route through Tittonia, Burgos and Tollodolid, a distance of more than 900 miles, by uninterrupted rail to Madrid, the Spanish center and capital. The whole route possesses especial interest, is rich in historic associations, and is not, like central Europe, flooded with Americans and English. You are left to mingle with and study the habits of a native population which has changed little for centuries.

At Orleans you find the memory of Joan of Arc, or the "Maid of Orleans," cherished. Her deeds are recorded in monumental stone, and bronze, in all the public squares and places; many paintings and engravings in the hotel and shop windows, bear testimony to the same fact.

Bordeaux is not only the largest town through which we pass between Paris and Madrid, and hence commercially most important, but also possesses more which claims attention from the medical observer than any other. In Bordeaux there are two large hospitals, one more especially for the citizens and another for strangers and commercial men. Each hospital has more than four hundred beds, is managed in the usual French method, both in the medical attendance and in the nursing, which latter is by the Sisters of Charity. There is, therefore, to one familiar with Parisian hospitals little which is novel; they are but copies of the parent model.

In the vault or "cavern" attached to St. Michael's church you are shown the preserved bodies of 70 to 100 individuals of all ages and both sexes. Their history, as furnished by the guide books and *valets de place*, is as follows: There is a belfry attached to the church which was built between 1472 and 1480, the steeple of which formerly rose to the heighth of 300 feet. The ground around this belfry was used as a church-yard or cemetery. In the early part of the French revolution the church-yard was closed, and the bodies dug up, and the belfry and steeple nearly razed to the ground. The bodies above referred to, many of which had been buried three or four hundred years, were found to be *perfectly preserved.* They look like mummies or dried fish, or half tanned leather. The age, sex, and even the features to a considerable extent can be made out. Here stands a General, who is known to have fallen in a duel, with the wound in the chest which caused his death, still gaping and patulous. Then an old woman, whose whole breast, and the surrounding tissues, were destroyed, probably by cancer, the remainder of the body being entire. Upon the other side are those who seemed to have died in full health, plump and round; there are others emaciated to skeletons, as by consumption. Among the number are two or three, who, from the positions which they have assumed, the twisting of the neck, tearing of the hands and the contortions of countenance manifest, leave little doubt on the mind of the beholder that the statement of the guide is correct, when he assures you that they were buried alive, these positions could only have resulted from their efforts to release themselves from a confinement most horrible to contemplate.

It may also be added that the drapery, the linen, the lace even with which the linen was ornamented; the hair, the beard, and its color are in many instances perfectly preserved. To my mind these bodies, thus completely *preserved*, (not converted into adipocere,) the liquid parts only having escaped by evaporation, present a curious subject of inquiry to the philosophic mind, as to the cause. What were the antiseptic qualities peculiar to this soil which enabled it thus completely to arrest the process of decomposition? So far as I am aware the case is without a parallel. From the guide and the gentleman to whom I had letters I could not learn that the subjects had ever been brought to the attention of the scientific investigator, and no suggestions as to the cause of this wonderful phenomenon had been made. Upon my return to Paris I propose to pursue the subject, when I shall, perhaps, find that it has already received such attention as in my opinion it demands, and if so your readers shall learn the benefit of any intelligence which I may be able to obtain upon this curious and interesting question. Meanwhile were I to hazard a guess, it would be that the soil in which those bodies were interred contained sulphate of iron, which by permeating the animal tissues completely arrested the ordinary process of decomposition, and must preserve them for an indefinite length of time. I am led to this suggestion simply from the color of the earth from which they were taken. But speculation is useless and I dismiss the subject for the present.

It may not be amiss to say that we paid our devotions to Bacchus by visiting the wine cellars or vaults, miles in extent, and testing such delicious specimens of their contents as our excellent friend submitted to our judgment.

Leaving for Boyone we passed through what are called the *Loudes*, immense pine barrens, as we should call them in America. Low, sandy plains, upon which nothing can be made to grow except small scrub pine, and here and there a vegetable sprout of the coarsest character, and pointed with a thorn or briar. A few sheep are here and there to be seen, the only product of the country, except pitch. They are guarded by a race of diminutive, half starved looking shepherds. These diminutive and short-lived beings, clothed in the skins of the animals they tend, may be seen upon high stilts, running hither and thither with great fleetness, guarding and bringing their flocks together, or leaning back upon a peg put into a pole which they carry, and with the stilts, forming a tripod, taking their scanty meal, or whiling the time away by knitting.

Arriving at Madrid in the middle of February we were not a little suprised to find a city situated in latitude about the same as New York city, and in a mountainous region, more than 2,300 feet above the sea, so warm as scarcely to need fires. Indeed it was difficult to find a room in the best hotels in which a fire could be built. The question of difference in temperature in the same latitudes in Europe and America has never been satisfactorily accounted for. There are, as you know, many theories, but to my mind they are insufficient to account for the fact, that, whilst you were shivering with the thermometer below zero, we, when in the north of France were reveling in *green* fields, in an atmosphere which has for years been constantly above the *freezing* point, although nearly *ten* degrees further north. Cherbourg is further north than Quebec, and as far as the center of Lake Superior.

Madrid contains about four hundred thousand inhabitants, is situated upon an elevated platteau in nearly the geographical center of the kingdom. When you bear in mind the migatory character of the "universal yankee nation," you will doubtless be surprised to learn that, aside from those who are connected with the legation, in a population of nearly half a million, there is but one resident American. Dr. Mackedean, a most intelligent and courteous gentleman, to whom we were under many obligations for courtesies during our stay, is a dentist of deserved celebrity. He has resided in Madrid more than twenty years, has been decorated and made dentist to the Royal family, and acquired for himself wealth and reputation. I am happy also in being able to add that the Doctor was a zealous supporter of the government of his native country during its late struggle, and kept the public mind in Madrid in a great degree informed as to the merits of our cause. It is not a little remarkable that the most eminent dentists in France, Spain and Portugal are Americans. This arises doubtless in a great measure from the fact that in European countries dentistry is simply studied and practiced as an art, whilst in America it has been elevated to a science, without diminishing the artistic skill exercised in the various manipulations of the art.

Medical education in Spain is under the control of the government. There have been medical schools and hospitals in Spain ever since its conquest by the Moors. They introduced Arabic lore and drugs, and established colleges, to which all Europe resorted for instruction. Surgery was neglected on account of the prohibition in the Koran to dissect. As early as the eighth century, however, a school of medicine was established in Cordova, the library of which contained more than 250,000 volumes. And

French, English and Italians came hither to study the art of healing of these "magicians," as these learned Arabians were called. After the expulsion of the Moors, the Catholic Kings employed Moorish and Jewish doctors, patronized the profession, and founded hospitals and schools in Seville and Granada. The one at Madrid, San Carlos, the most important at this time, was not founded until 1783, by Charles III. It has a fine anatomical museum, has a faculty containing no less than sixteen professors, who are appointed and paid by government. There are in attendance upon the present course, about 800 students, being much the most flourishing school now in the kingdom. The standard of education preliminary to commencing the study of medicine is fearfully low in Spain, and has a decided tendency to degrade its practice into a mere art, and greatly lessens the respect in which the profession is held in the community. Only one in four of the whole population can either read or write, according to the best statistics available. With this absence, or nearly so, of popular education, any youth may attend the medical lectures without fee, and present himself as a candidate for the highest medical honors of the University without any investigation being instituted as to his previous attainments. True to traditionary association I am credibly informed that by far the largest number of recruits is derived from the barbers and their apprentices. To show that this assertion is not a mere poetic fiction, founded upon no better authority than historic association with the profession in its infancy, I will add that, the accoucheur to Her Majesty in her recent confinement, began life as a practical barber, and that the two most celebrated practitioners now in Madrid can boast of having been apprentices to him, and following his example now abandoned the razor and strop for the scalpel and lancet, and thus "hewed" their way to their present exalted position. The trade of the barber, not only in Madrid, but in the rural cities, serves the same end with the Spanish medical student, which school teaching has accomplished for so many young Americans. Perhaps no one circumstance could be mentioned which would better illustrate the difference not only in the road taken to professional advancement, but in intelligence of the Spanish as compared to the American people. Madrid is well supplied with hospitals, affording abundant opportunity for clinical instruction to the medical student. There are two large general hospitals and several special hospitals for venerial and other diseases, and a large military hospital. The latter was built in 1841, contains 600 beds, and is in many respects a model hospital. The amount of air space allowed to each patient is one thousand cubic feet, and ventilation is well secured by basement openings. The diet-table is generous, and the whole arrangements for the care of the sick and wounded, are such as would do credit to the best of our military hospitals. Upon inquiry I found that in all capital operations they are in the habit of using chloroform, and also that it was used by the obstetrician in surgical midwifery. Ether had been for a time resorted to, but had been entirely superseded by chloroform, in the conviction that the latter was equally safe and far more reliable. There is in this institution a bed or ambulance-couch for transporting patients from room to room, or from the operating table to the bed, which was superior to anything I have elsewhere seen, and I will endeavor to procure a model for the benefit of our Buffalo hospitals. Before leaving this institution attention should be called to the room or *salle* for the treatment of the diseases of the eye. It was a fine, lofty chamber, 150 feet by 30, and at least 25 feet high, and so constructed as to exclude light entirely; or only to admit it through a green medium, and in such amount as might be prescribed. In this ward ventilation and cleanliness were perfect. I do not mean to be understood as saying, by any means, that all ophthalmic diseases require the exclusion of light for their proper treatment, but in the cases, if any there may be, where it is necessary to keep the patient in a dark chamber, the arrangements of this ward are universally perfect. In other departments of this immense institution are depots of supplies of surgical instruments, medicines, beds, bedding, etc., and arranged in readiness for immediate transportation to any part of the kingdom as the exigencies of the army may require.

In Madrid the status of the profession, as a whole, is by no means what it should command. Fees are low, except as gratuities from the rich and liberal; bickerings, jealousies and cliques among its members as prevalent here as I regret to admit they are elsewhere. In the rural districts and smaller cities the practice of the time-honored *sangrado* still prevails, and the non-progressives in America are by these venerable practitioners abundantly sustained in the free resort to the lancet and in the frequent administration of heroic doses, to the utter neglect of the patient's sustentation, aud setting at defiance all hygienic rules. Bleeding, blistering, calomel and starvation are the remedies to which the routinists subject their patients, with an energy which renders the situation of the unlucky individual anything but safe or desirable. Reformation and a more physiological and rational appreciation of the curative efforts of nature, which is the prominent feature in the advances of the profession at the present day, begin to manifest their efforts in Spain, and must sooner or later triumph over prejudice and ignorance.

The picture gallery in Madrid is scarcely second to any in the world. It is particularly rich in the old masters, though by no means deficient in the modern schools. There are no less than 10 original Raphaels, 46 Murillas, 43 Titians, 64 Valesquezs, 34 Tintonelas, 62 Reubens, 52 Teniers, 37 Van Dycks, 40 Rembrants, besides numberless others of scarcely less merit. The *Armoria* will claim the attention of all Americans, if for no other reasons than to see the "Authentic armor worn by Christopher Columbus." It weighs 41 pounds. No. 2342, which stands near by, in the same museum, is the equestrian armor of Cortez.

We were fortunate in being in Madrid during the last week of carnival. There is perhaps no place at the present time in Europe, where so much attention is given to costume, masking and festivity during this season as in the Spanish capital. Paris, Rome and Florence are now so filled with Americans and English as completely to change their nationality, and convert the hotels and fashionable boarding houses and promenades and places of amusement to their own uses, where you meet few who do not speak the English language. In Madrid carnival is the gayest season in the year, and the tourist will not fail to resort to the Prado during the then last days of its duration, which are three glorious days of public merriment. Half the population turns out *de mascora* to "intrigue" and "chaff" the other half; all in the pleasantest and most good humored way. Upon the last day of these festivities the Prado, the public promenade and drive of Madrid, presented a scene of indescribable gaiety. The maskers, in every conceivable disguise and costume, and of both sexes, many on foot, some on horseback, and others in carriages, occupied the center of this long and broad avenue. The lookers on who could "sport a carriage," were privileged to fall into line and drive around this gay multitude, thus forming a circle of carriages several miles in length, enclosing this brilliant assembly, whilst the great mass of the population occupied seats or walked about in the large, open grounds upon either side of the lines of carriages. It would be impossible for me to give any adequate idea of this magnificent spectacle, this pandemonium turned loose. It is the great fete of the Catholic year, and is entered into with a gist and merriment which to an American is utterly incomprehensible.

In justice it should be mentioned as a characteristic of this people, that with all this "abandon" in this immense assemblage, there was to be seen no drunkenness. Indeed it is remarkable that in all their popular outbursts, at fetes, bull fights, races, or elsewhere, drunken men, black eyes, blacklegs and blackguards are never to be seen, and all and each observe a dignified deportment and pay great respect to authority. Without attempting to afford a rationale to a condition so unlike what we behold in America under similar circumstances, I will close my rambling letter and subscribe myself,

Yours, ever, JAMES P. WHITE.

